# Application of Deep Learning Models for Automated Identification of Parkinson’s Disease: A Review (2011–2021)

**DOI:** 10.3390/s21217034

**Published:** 2021-10-23

**Authors:** Hui Wen Loh, Wanrong Hong, Chui Ping Ooi, Subrata Chakraborty, Prabal Datta Barua, Ravinesh C. Deo, Jeffrey Soar, Elizabeth E. Palmer, U. Rajendra Acharya

**Affiliations:** 1 School of Science and Technology, Singapore University of Social Sciences, Singapore 599494, Singapore; hwloh002@suss.edu.sg (H.W.L.); cpooi@suss.edu.sg (C.P.O.); 2 Cogninet Brain Team, Cogninet Australia, Sydney, NSW 2010, Australia; wanrong@cogninet.com.au (W.H.); prabal.barua@usq.edu.au (P.D.B.); 3 Faculty of Engineering and Information Technology, University of Technology Sydney, Sydney, NSW 2007, Australia; subrata.chakraborty@uts.edu.au; 4 School of Business (Information Systems), Faculty of Business, Education, Law & Arts, University of Southern Queensland, Toowoomba, QLD 4350, Australia; jeffrey.soar@usq.edu.au; 5 School of Sciences, University of Southern Queensland, Springfield, QLD 4300, Australia; ravinesh.deo@usq.edu.au; 6 Centre of Clinical Genetics, Sydney Children’s Hospitals Network, Randwick, NSW 2031, Australia; elizabeth.palmer@unsw.edu.au; 7 School of Women’s and Children’s Health, University of New South Wales, Randwick, NSW 2031, Australia; 8 School of Engineering, Ngee Ann Polytechnic, Singapore 599489, Singapore; 9 Department of Bioinformatics and Medical Engineering, Asia University, Taichung 413, Taiwan; 10 Research Organization for Advanced Science and Technology (IROAST), Kumamoto University, Kumamoto 860-8555, Japan

**Keywords:** Parkinson’s disease (PD), deep learning, computer-aided diagnosis (CAD), SPECT, PET, MRI, EEG, gait, handwriting, speech

## Abstract

Parkinson’s disease (PD) is the second most common neurodegenerative disorder affecting over 6 million people globally. Although there are symptomatic treatments that can increase the survivability of the disease, there are no curative treatments. The prevalence of PD and disability-adjusted life years continue to increase steadily, leading to a growing burden on patients, their families, society and the economy. Dopaminergic medications can significantly slow down the progression of PD when applied during the early stages. However, these treatments often become less effective with the disease progression. Early diagnosis of PD is crucial for immediate interventions so that the patients can remain self-sufficient for the longest period of time possible. Unfortunately, diagnoses are often late, due to factors such as a global shortage of neurologists skilled in early PD diagnosis. Computer-aided diagnostic (CAD) tools, based on artificial intelligence methods, that can perform automated diagnosis of PD, are gaining attention from healthcare services. In this review, we have identified 63 studies published between January 2011 and July 2021, that proposed deep learning models for an automated diagnosis of PD, using various types of modalities like brain analysis (SPECT, PET, MRI and EEG), and motion symptoms (gait, handwriting, speech and EMG). From these studies, we identify the best performing deep learning model reported for each modality and highlight the current limitations that are hindering the adoption of such CAD tools in healthcare. Finally, we propose new directions to further the studies on deep learning in the automated detection of PD, in the hopes of improving the utility, applicability and impact of such tools to improve early detection of PD globally.

## 1. Introduction

The purpose of this systematic review is to provide a comprehensive review of automated Parkinson’s disease (PD) detection using deep learning models, and to further promote deep learning models as a potential computer-aided diagnostic (CAD)-based tool for clinical decision support systems. In Section 1, we introduced the background of PD, the limitation of the current diagnostic method, and the CAD tool being a possible solution to alleviate the burden of neurologists. Thereafter, we elaborated on the benefit of deep learning models over machine learning models as a CAD tool and illustrated the mechanics of the two most popular types of deep learning models: convolutional neural network (CNN) and long short-term memory (LSTM). Section 2 describes the adoption of the PRISMA model for the systematic review of automated PD detection studies using deep learning models. To build the systematic review, a total of 63 studies were chosen after a systematic removal of the irrelevant studies. In Section 3, these studies were then split into two categories: brain analysis and motor symptoms. Subsequently, data analysis and visualization were performed for each category. In Section 4, we also discussed the current trend observed from the 63 research studies, the limitations of deep learning models for CAD detection, and presented the proposed directions for future work which can increase the adoption of deep learning models as a CAD tool. Finally, Section 5 concludes the review by summarizing the key findings, limitations, and the potential of deep learning models as a CAD tool to support clinical decisions.

### 1.1. Background

PD is an incurable neurological disease that results in progressive deterioration within the central nervous system and debilitating neurological symptoms [1]. The underlying cause of the neurodegeneration in PD is still partially understood, but key pathophysiological features are the gradual loss of dopaminergic neurons in a part of the midbrain known as substantia nigra pars compacta (SNpc), and the accumulation of misfolded alpha-synuclein protein in ‘Lewy bodies’ within the cytoplasm of neuronal cells in several different brain regions [2]. The dopaminergic pathway between the SNpc and the dorsal striatum, also known as the nigrostriatal pathway, is critical for movement control. Hence, disruption to the nigrostriatal pathway results in motor abnormalities in affected individuals with PD, including tremors, rigidity, and bradykinesia [3]. Affected individuals also experience non-motor symptoms, including constipation, depression, sleeping disorders, and reduction of smell [1,3].

Between 1990 and 2016, the number of people diagnosed with PD had doubled from 2.5 million to 6.1 million. This means the age-standardized prevalence rate increased by 21.7% [4]. Hence, PD is one of the most prevalent neurological disorders, with immense societal impacts, yet no curative treatments [5]. The gold standard treatment for PD is the dopamine precursor amino acid levodopa, which, in the initial stages of PD at least, can alleviate many motor symptoms by substituting for striatal dopamine loss [6]. However, its use can be complicated by the development of motor complications, including drug-induced dyskinesias, and patients also have L-DOPA-resistant motor features including treatment-resistant tremor, postural instability, swallowing and speech disorders [2]. A range of modifications of dopaminergic treatments, as well as non-dopaminergic pharmacological therapies and non-pharmacological treatments such as deep brain stimulation, may be required over time. Rehabilitation and psychosocial supports are also key to try and maintain affected individuals’ quality of life, and thus early diagnosis to allow instigation of expert multidisciplinary care is a key priority. Moreover, novel therapies that may actually modify the underlying disease processes are the goal of a large body of global research: it is likely that such advanced therapeutics, such as gene therapy, will need to be instigated as soon as possible in order to have maximal effect, as has been found to be the case for other degenerative conditions such as spinal muscular atrophy [7]. Therefore, early diagnosis is especially crucial in the optimal current and future management of PD, to ensure maximal functional outcomes for affected individuals. 

At present, the diagnosis of PD is based on core clinical features, and the accuracy of clinical diagnosis can be improved by following standard clinical criteria, such as the UK Parkinson’s Disease Society Brain Bank (UKPDSBB) [8], such as the presence of bradykinesia and absence of certain exclusion criteria. This clinical criteria rely on the expertise of a neurologist, but still are flawed: for example the diagnostic accuracy using the UKPDSBB, even in specialist neurology centres, is only slightly above 80%, compared to post-mortem pathological examination as gold standard [9]. Moreover, there is a global shortage of neurologists, especially in countries experiencing aging populations where there is a high frequency of neurological disorders [10]. This increases the waiting time for affected individuals to get diagnosed with PD. As a consequence, 60% of the dopaminergic neurons are typically lost by the time of diagnosis [2]. 

In efforts to meet the healthcare demands, there are interest in the possibility of using CAD tools based on artificial intelligence methods, namely machine learning (which potentially involves the more conventional pattern recognition approaches) or deep learning (which may involve sophisticated multi-layered neuronal systems), to perform an automated diagnosis of PD [11,12,13]. These CAD tools can perform automated detection using the biomarkers of PD, such as Electroencephalogram (EEG) signals, posture analysis in the gait cycle, voice aberration, or brain imaging such as Magnetic Resonance Imaging (MRI) and Positron Emission Tomography (PET) [14]. In a conventional machine learning model, it is mandatory to extract the features from the biomarkers and then select the most salient features in order to train the model [15,16,17,18,19]. This is a required step because machine learning models by itself are not capable of learning the high dimensional data in their raw forms, otherwise, the model is likely to overfit the dataset [20]. Also, the selection of the most relevant features must be carried out by an experienced expert system that is knowledgeable in terms of various feature selection tools [15,16]. This has led to the somewhat poor adoption of machine learning models as the future CAD tools as feature extraction and selection can be complicated procedures comprehensible by machine learning experts, but not so by the end-user of the CAD tool [21,22]. Such end-users may involve healthcare experts such as practicing clinicians, health researchers, or other domain applications. 

**Figure 1:**
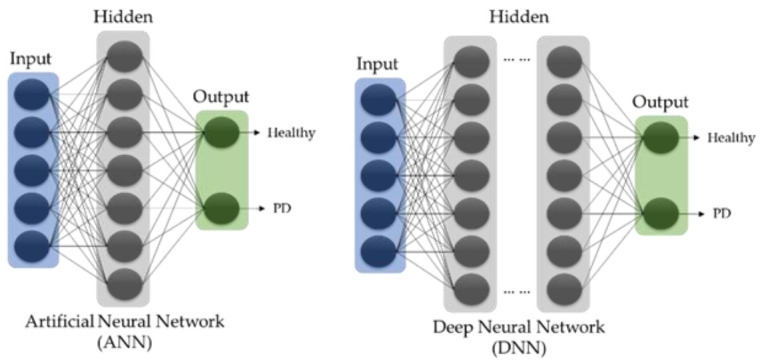
Basic architecture of ANN and DNN models.

Deep learning models, which are of increasing interest with big data and can resolve some of the limitations of machine learning models by eliminating the need for feature selection, feature extraction tools. Such models are capable of learning the high-dimensional data, and they may function analogously to the neurons in the human brain [23]. The conventional forms of machine learning models known as artificial neural networks (ANN) consist of three main layers: the input, the hidden, and the output layer as shown in Figure 1. All three layers within a neural network contain artificial neurons that are interconnected, as denoted by the black lines. As the neural network learns via a learning algorithm (e.g., backpropagation), the weights of the connection (black lines) between the neurons update iteratively [23]. The neurons, which act as an individual classifier, determines the output signal after processing the weights from its previous connections [23]. 

When an ANN model has been constructed into an architecture that has more than one hidden layer, the system is then known as deep neural networks (DNN), and such systems are then capable of learning the data with a higher degree of complexity [23] (Figure 1). In deep learning algorithms, there are often other classes of model, such as CNN, recurrent neural network (RNN), and LSTM, that utilize DNN as their basic principal architecture. 

### 1.2. Convolutional Neural Network (CNN)

**Figure 2:**
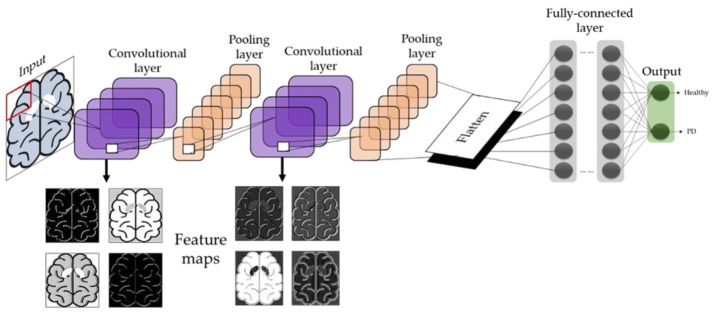
Basic architecture of the CNN model.

In any CNN model, the input layer of a typical DNN model is replaced by a series of convolutional and pooling layers, as also shown in Figure 2. If DNN is described as the neurons in our brain, then the CNN may be considered as the human visual system [24]. The first convolutional layer contain numerous filters which extract features from the input image to generate multiple feature maps. The subsequent pooling and convolutional layers reduce the dimension of the feature maps and further enhance the features, thereby reducing the complexity of the feature map and the likelihood of overfitting [25]. This could be considered as analogous to the human visual system, where the visual cortex attempts to break down images into simpler representations for the brain to perceive the image with ease [24]. 

After the final pooling layer, the feature maps are converted into single-list vectors at the flatten layer (Figure 2). The neurons in the neural networks, also known as the fully connected layers, will then learn to recognize the features from the single-list vectors and perform image classifications [25]. Hence, CNN models are known for their exemplary image recognition ability, which many studies have successfully demonstrated the success of CNN in medical imaging, including the recognition of breast tumors, and eye diseases using mammogram and color fundus images, respectively [26]. Apart from medical images, CNN has also demonstrated success in biometric face recognition systems for human tracking purposes [27,28].

### 1.3. Long Short-Term Memory (LSTM)

The LSTM model is an improvement from its predecessor methods known as RNN [16]. Just like its name suggests, the LSTM model attempts to mimic how the brain stores memories and makes predictions based on immediate past events stored in the memories [24]. Both the RNN and the LSTM models are known for their ability to recognize patterns in sequential data [16]. However, the vanishing gradient has often been a very common problem in RNN models, where a large information gap exists between the new and old data, causing erroneous signals to vanish during the model’s training phase. As a result, the RNN model is not able to learn the data that has long-term dependencies. Hence, the LSTM model has been developed to resolve the problems of vanishing gradient in RNN models [29]. 

**Figure 3:**
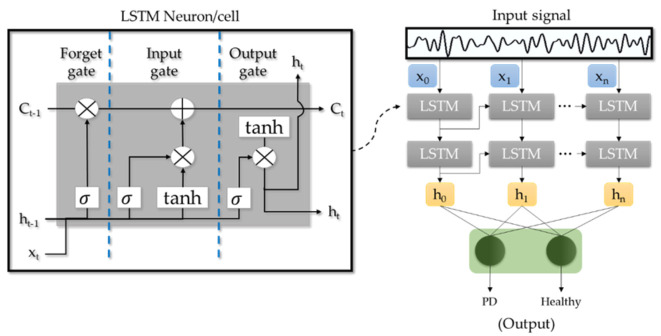
Basic architecture of the LSTM model.

The neurons in a typical LSTM model adopt a unique gate structure [30] denoted as the forget gate, input gate, and output gates (Figure 3). The input gate decides if the new information (x_t_) should be stored in the cell, the output gate decides what information should output as the hidden state (h_t_), and the key to eliminating the vanishing gradient problem lies in the forget gate [30,31]. The sigmoid (σ) function in the forget gate is used to deduce if the information brought from the previous cell state (C_t−1_) should be kept or forgotten, thereby removing irrelevant data, and reset the information in the cell appropriately [30,31]. This prevents large discrepancies between the old and new information that will eventually lead to vanishing gradient problems. In addition, useful information continuously gets backpropagated in the LSTM model, allowing it to memorize patterns in long-term dependencies [30,31]. Hence, the strong pattern recognition ability of LSTM models is widely implemented in applications such as speech and handwriting recognition [32,33]. LSTM models are also suitable in forecasting stock prices in financial markets which are dynamic and non-linear in nature [34,35]. 

## 2. Materials and Methods

**Figure 4:**
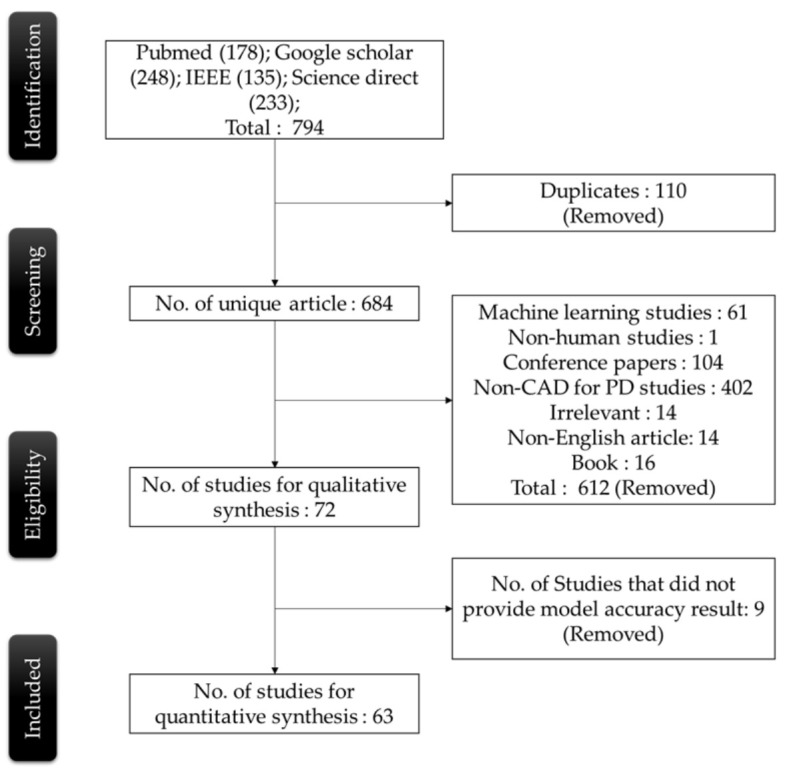
Flow diagram of the PRISMA model in the article selection process to build the systematic review.

This systematic review applied the PRISMA model [36] to analyze the most relevant studies on PD detection using deep learning models from the period January 2011 to July 2021. All the resources were systematically searched through PubMed, Google Scholar, IEEE, and Science Direct using the Boolean search strings, as shown in Table 1. A total number of 794 studies that contained these Boolean search strings were identified, which also included 178 studies from PubMed, 248 studies from Google Scholar, 135 studies from IEEE, and 233 studies from Science Direct. From the 794 articles initially identified, a total of 110 duplicate studies were removed. After this, a total of 612 articles (61 traditional Machine Learning studies, one non-human study, 104 conference papers, 402 Non-CAD for PD studies, 14 irrelevant studies, 14 non-English articles, and 16 books) were also excluded according to their relevance with this review. Eight studies were further removed from the list as they did not provide model-accuracy results. The final number of research studies that qualified for inclusion in this review was set to 63. Figure 4 shows a detailed process of the PRISMA method in the selection of the most relevant articles.

## 3. Results

There are two parts to this section. Section 3.1 Brain analysis covers 23 deep learning studies performed on Single Photon Emission Computed Tomography (SPECT), PET, MRI, ultrasound, and EEG. Section 3.2 Motor symptoms covers 40 deep learning studies performed on gait, handwriting, speech, Electromyogram (EMG), and other movement-related tests. The details of the deep learning studies under brain analysis and motor symptoms categories are in Appendix A Table A1 and Table A2, respectively.

### 3.1. Brain Analysis

**Figure 5:**
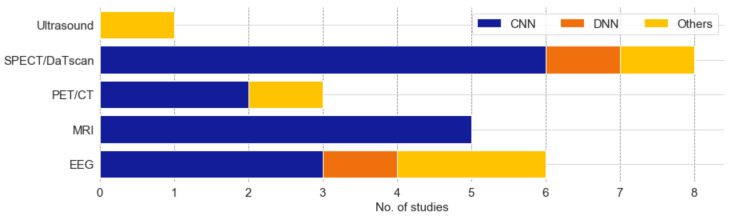
Stacked bar plot of the number of deep learning models proposed for each modality of brain analysis.

**Figure 6:**
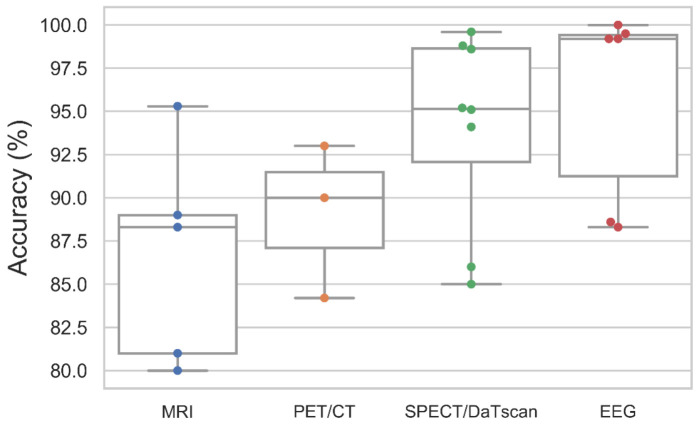
Box and whiskers plot of the model accuracy of deep learning studies using various modalities of brain analysis.

MRI, PET, and SPECT are the common brain imaging modalities used to diagnose PD. The public image dataset for these three imaging modalities can be downloaded from Parkinson’s Progression Markers Initiative (PPMI) database (https://www.ppmi-info.org/, accessed on 12 October 2021). Numerous studies in Appendix A Table A1 had attempted to develop deep learning models to distinguish the brain of PD patients from healthy controls. Among them, a majority of the studies had chosen SPECT images to train their deep learning models; 8 studies used SPECT images, 5 studies used MRI images, and 3 studies used PET images (Figure 5). Studies that had used SPECT images for automated PD detection also achieved a higher model performance, as compared to MRI and PET images (Figure 6). This may be because DaTscan is used for SPECT imaging. DaTscan is the name of the radioactive tracer, ioflupane (I123), that is specifically used to detect dopamine transporters in the brain [37]. Hence, it can better represent the loss of dopaminergic neurons in the PD brain [38]. On the other hand, the radioactive tracer used in PET for PD diagnosis is known as ^18^F-FDG, which is primarily used to assess neuronal function via regional cerebral glucose metabolism [39].

**Figure 7:**
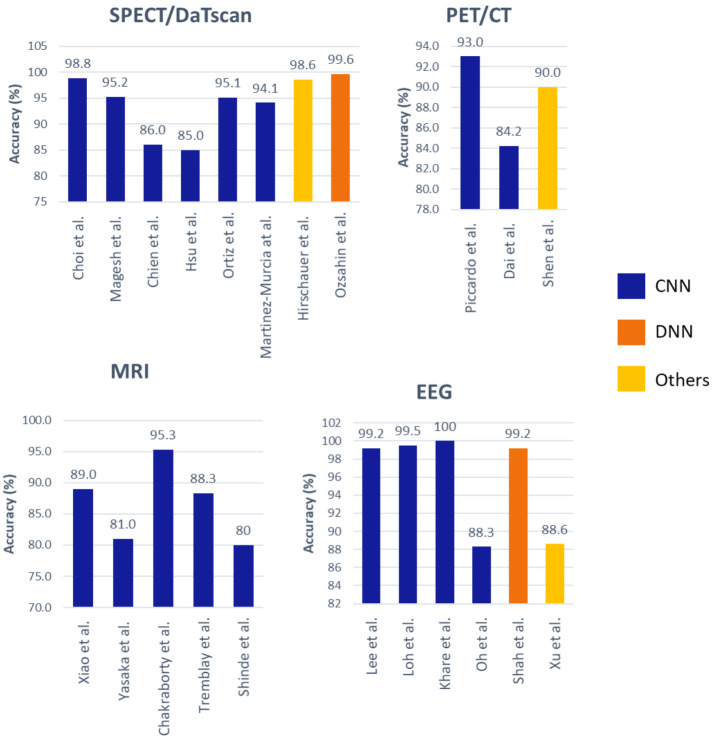
Bar plot representation of the model accuracy by various investigators for different modalities of brain analysis.

A majority of the studies that focused on image analysis proposed CNN models for an automated detection of PD (Figure 5). For the case of SPECT imaging, the highest performing CNN model was developed by the study of Choi et al. [37], which had evaluated their proposed model (i.e., PD net) with two datasets: the PPMI dataset, which obtained an accuracy of 96%, and a private dataset (SNUH cohort) with an accuracy of 98.8% (Figure 7, Appendix A Table A1). Both results exceeded the performance of two human raters whose accuracies were 90.7% and 84% each for the PPMI dataset. There was only one study by Ozsahin et al. [40] that has proposed a back-propagation neural network (BPNN), which achieved the highest model accuracy of 99.6% using the binarized image of SPECT images (Figure 7, Appendix A Table A1). However, the applicability of the CNN model has been advocated in a majority of studies in SPECT imaging (Figure 5). In any event, we aver that for practical and ethical purposes, the suitability of the CNN or the BPNN model for SPECT imaging should still be assessed via clinical trials. As for the PET and the MRI study cases, we note that the highest performing CNN model was 93% [41] and 95.3% [42], respectively (Figure 7, Appendix A Table A1). 

To date, only the study of Shen et al. [43] had attempted to use ultrasound, namely transcranial sonography (TCS) images for automated PD detection (Appendix A Table A1). They proposed a deep learning model known as Multiple kernel mapping—broad learning system (MEKM-BLS) that has a wider feature and enhancement node/neurons than a typical DNN model. This method has the ability to map the features from the feature node directly onto the enhancement node. However, their model only achieved an accuracy of 78.4%, lower than that of MRI, PET and SPECT. Nonetheless, ultrasonography has several advantages such as low cost, fast, and does not have radiation exposure [44]. Furthermore, a study by Mehnert et al. [44] demonstrated that interpretation of TCS for PD diagnosis can reach a sensitivity score of 95% by experienced sonographers. Hence, there is room for improvement for ultrasonography in automated PD detection, and future work to implement CNN models for the interpretation of TCS images should be considered. 

Apart from brain imaging issues, the physiological signals such as the EEG can also reflect brain abnormalities that are unique to the prevalence of PD [45]. This aspect has been reported, particularly that the EEG frequency of a PD patient is abnormally slow, compared to that of a healthy individual [46]. In this review, we have found 6 studies that had proposed deep learning models to recognize EEG characteristics for automated detection of PD. Nearly half of these studies proposed the use of the CNN model [25,47,48], and the remaining three studies had proposed the application of an RNN [49], DNN [50], and a hybrid deep learning model that combines CNN and RNN algorithms [51] (Figure 5). The best-performing model was developed by Khare et al. [47], who has also proposed a CNN model with smoothed pseudo-Wigner Ville distribution (SPWVD) features from EEG signals as an input, and further obtained an accuracy near 100% (Figure 7, Appendix A Table A1). This shows that CNN models are likely to achieve a high classification accuracy for one-dimensional data such as EEG signals. Like the data of ultrasound tests, the EEG data are somewhat cheaper and offer a low-risk alternative to the MRI, PET, and SPECT datasets, but unlike ultrasound, the overall accuracy of studies that implemented EEG signals (95.8%) is on par with studies that have used SPECT images (94.1%) (Figure 6). 

### 3.2. Motor Symptoms

**Figure 8:**
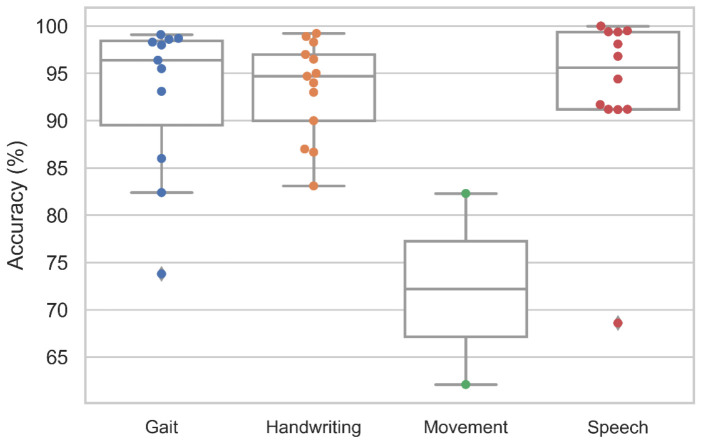
Box and whiskers plot of the model accuracy of deep learning studies using various modalities of motor symptoms.

Since PD is characterized by involuntary motor control, an assessment of motor can be utilized for the diagnosis of PD. Such assessments could include gait, handwriting, speech, and other movement-related tests as illustrated in Figure 8. 

**Figure 9:**
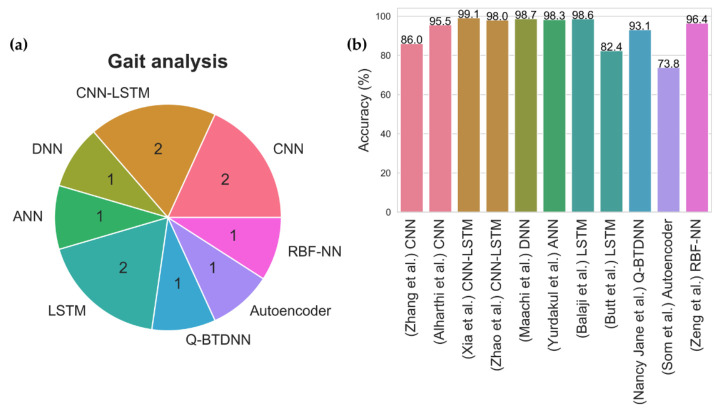
(**a**) Pie chart representation of various deep learning models proposed for gait analysis and (**b**) Bar chart representation of model accuracy for each deep learning study in gait analysis.

In principle, Gait refers to the walking patterns of an individual. In the case of PD, the body’s stiffness and postural instability may worsen as the disease progresses, leading to gait disturbance [52]. In this respect, the gait features can be utilized to train deep learning models in the detection of PD. The key features of gait include kinetic features such as ground reaction force (GRF) and kinematics features such as stance and swing phase of the foot [52]. There are currently 11 deep learning studies that have attempted to analyze the gait for PD detection, and a wide variety of deep learning models have thus been proposed (Figure 9, Appendix A Table A2). Among them, two studies while proposing a set of hybrid models by combining the CNN and LSTM model have achieved a high overall accuracy [53,54] (Figure 9). The best-performing hybrid CNN-LSTM model was also proposed by Xia et al. [53], using vertical GRF at multiple points of time during the gait cycle. The idea of implementing a hybrid CNN-LSTM model for gait analysis is to have the CNN layer extract the salient gait features, and the LSTM layer to analyze the temporal pattern of the gait features in a walking cycle. As a result, Xia et al. [53] achieved the highest model accuracy of 99.1% (Figure 9, Appendix A Table A2), using a dataset that came from three research groups: [55,56,57]. Similarly, two other studies that had proposed DNN [58] and LSTM [59] model also achieved high-performance results that are on par with the CNN-LSTM model (Figure 9, Appendix A Table A2). Hence, future deep learning studies based on gait analysis could focus on the development and implementation of these three models. 

**Figure 10:**
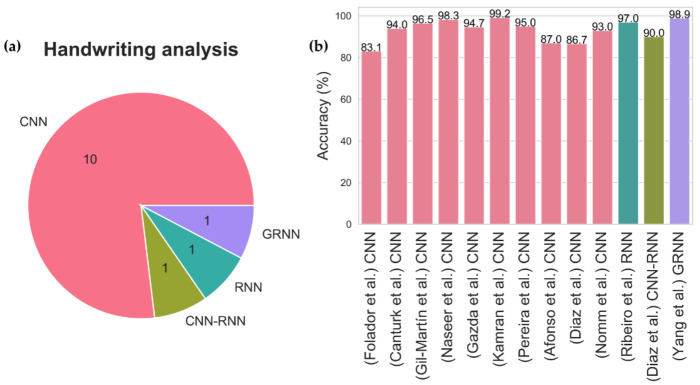
(**a**) Pie chart representation of various deep learning models proposed for handwriting analysis and, (**b**) Bar chart representation of model accuracy for each deep learning study in handwriting analysis.

The deterioration of handwriting ability is another telltale symptom of PD, and this is often seen in a majority of PD patients but is not included as a diagnostic criterion of PD [60]. A PD patient may exhibit abnormally small handwriting, termed micrographia, due to rigidity and tremors in the writing arm [61]. Thirteen studies on deep learning algorithms have attempted to diagnose PD using handwritten drawings with one of the three common PD handwriting datasets: PaHaW dataset [62], HandPD [63], and NewHandPD [64]. All three datasets involve a series of drawing and writing tests, and one of the common tests that exist in all three datasets is the spiral drawing test. Similar to the brain imaging, most studies had proposed using CNN models to differentiate handwritten drawings of PD patients from healthy controls (Figure 10). The best performance was achieved by Kamran et al. [65] who has tested the six common transfer learning architecture of CNN, namely AlexNet [66], GoogleNet [67], VGGNet-16/19 [68], and ResNet-50/101 [69]. These transfer learning models have been previously trained using a well-known image dataset known as ImageNet which consists of more than 1 million images. Kamran et al. [65] then fine-tuned the transfer learning models to adapt to the handwritten drawings of PD and healthy controls, and the highest model accuracy was achieved by AlexNet [66] with 99.22% (Figure 10, Appendix A Table A2). 

Only two studies have to far attempted to use a small-scale movement-related test like swallowing [70] and finger tapping [71] (Appendix A Table A2). These two studies had proposed different deep learning models each, and the best performance of 82.3% was achieved by Jones et al. [70], using an ANN model with video-fluoroscopic and manometric data collected from the boluses which were delivered to the subject’s oral cavity using a syringe. Videofluoroscopic data includes information like laryngeal, hyoid, and epiglottic movement, while manometric data includes information such as rise time and rate of the velopharynx and mesopharynx. 

**Figure 11:**
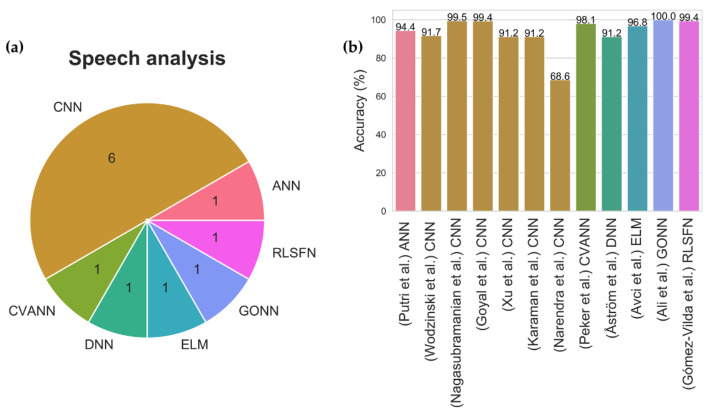
(**a**) Pie chart representation of various deep learning models proposed for speech analysis and, (**b**) bar chart representation of model accuracy for each deep learning study in speech analysis.

Besides the visible movement disorder, the muscle control of speech is also affected in PD [72]. As a consequence, people with PD will experience voice abnormalities such as lower voice volume and slurred speech [72]. There are currently twelve studies that had attempted to use voice aberration to diagnose PD (Figure 11, Appendix A Table A2). A wide variety of deep learning models were proposed with half of these studies being on CNN models (Figure 11). Two of the CNN models were seen to achieve a high model accuracy of 99.5% [73] and 99.4% [74] (Figure 11, Appendix A Table A2). However, the best performing model was developed by Ali et al. [75] who proposed a genetically optimized neural network (GONN) with a model accuracy of 100% (Figure 11, Appendix A Table A2). At present, more studies had supported CNN model for speech analysis. Nonetheless, it should be noted that clinical trials are required to further justify if GONN or CNN is a better alternative for speech analysis. 

Like the analysis of the brain, motor symptoms of PD can also be assessed by physiological signals, namely EMG. However, only one deep learning study has attempted to use EMG for PD diagnosis with the ANN model [76], and the performance of their proposed model was 71%, less than that of the studies that focused on gait, handwriting, and speech (Appendix A Table A2). Hence, for EMG to be recognized as a potential biomarker for PD diagnosis, more research in this area is required. Otherwise, datasets such as handwriting and speech recordings, which have easier data collection procedures, are better alternatives than EMG. 

Lastly, two studies did not limit themselves to only one type of modality (Appendix A Table A2). The study of Vasquez-Correa et al. [77] used three input signals—speech, handwriting, and gait—for multimodel analysis of PD using the CNN model and achieved 97.6% accuracy. Oung et al. [78] used two input signals based on speech and motion data derived from wearable sensors to propose an extreme learning machine (ELM) for the detection of PD. Their ELM model architecture is similar to an ANN model whereby there is only one hidden layer in its network but the training process of an ELM differs from the ANN model. Basically, the ELM model only requires a single iteration for model training through a random selection of the most optimal hidden neurons, which results in a much faster training time and a lesser overfitting problem compared with the ANN model [79]. The model accuracy of ELM obtained by the study of Oung et al. [78] was 95.9%, and this figure is comparable to the accuracy of the CNN model proposed by Vasquez-Correa et al. [77] (Appendix A Table A2). Based on a synthesis of these information, we conclude that deep learning models that are also capable of multimodel analysis of PD, may be a useful practical tool for neurologists. In the future, as more clinical information and particularly the detailed and correctly labelled electronic datasets are available, deep learning models may further aid in the diagnosis of PD. Hence, future studies on deep learning should perhaps consider using multiple types of input signals for PD detection, instead of relying on just a single modality.

## 4. Discussion

There are five parts to this section. Section 4.1 provides the summary of results gathered from the previous section. Section 4.2 discusses the challenges that are affecting the adoption of CAD in healthcare. Section 4.3 provides solutions to tackle the challenges highlighted in Section 4.2 and Section 4.4 describes the future vision of the CAD tool in the diagnosis of PD with Section 4.5 listing down the limitations of this review. 

### 4.1. Result Summary

**Figure 12:**
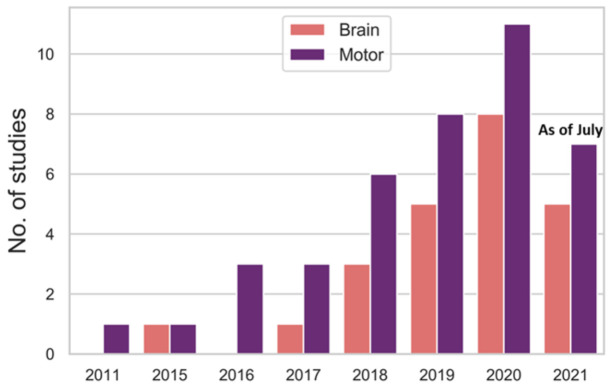
Bar chart representation of the number of deep learning studies published between January 2020 and July 2021 for brain analysis and motor symptoms.

**Figure 13:**
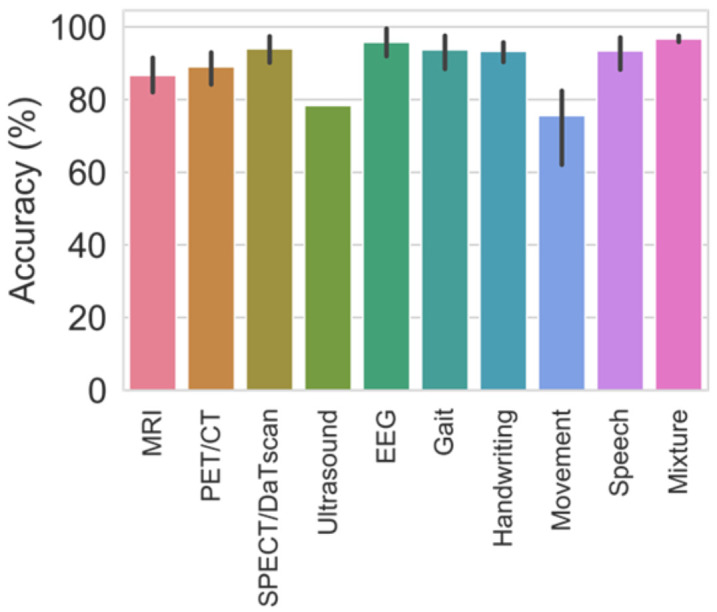
Bar chart representation of the average model accuracy from various deep learning studies obtained for each modality.

The application of deep learning models as a CAD tool for automated diagnosis of PD have been gaining popularity over many years. From Figure 12, the number of deep learning studies as of July 2021 has reached 12, which is more than half of the studies in 2020 (18 studies). Hence, it is very likely that the number of studies by the end of 2021 will exceed that of 2020. Every year, the number of deep learning studies bases on motor symptoms exceed that of brain analysis (Figure 12). This might be due to the ease of data acquisition for motor symptoms as the collection of data is less complicated than brain analysis and most of the datasets are publicly available. The overall model performance achieved by deep learning studies in each modality is favorable, especially for common modalities like MRI, PET, SPECT, EEG, gait, handwriting, and speech, which overall model accuracy had all exceeded 80% (Figure 13). 

**Figure 14:**
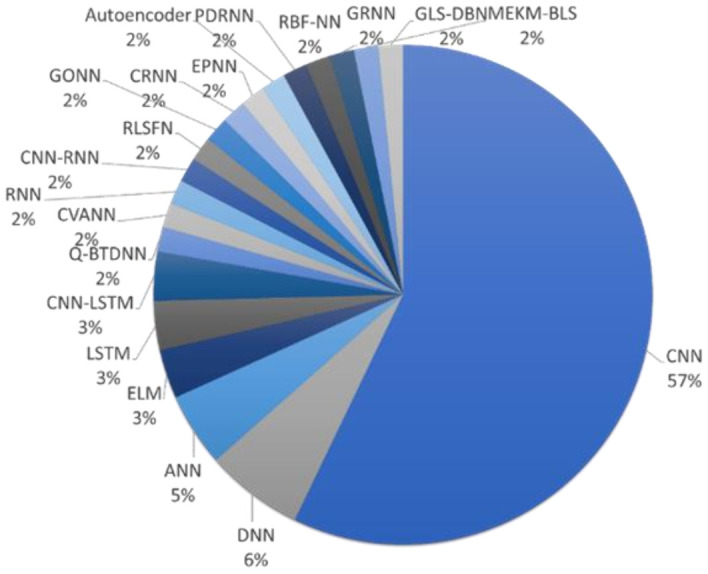
Pie chart representation of various deep learning models proposed for automated PD detection studies in this review.

This review underscores the following key aspects of the current deep learning studies for automated PD diagnosis:

- Deep learning models proposed by various studies have achieved a high predictive accuracy for the diagnosis of PD (Figure 13).
- About 57% of the deep learning studies for automated PD detection had proposed using the CNN model (Figure 14).
- CNN models have demonstrated to have high prediction accuracy for image classification such as brain imaging (SPECT, PET, and MRI), and handwriting recognition.
- Our results have also shown that CNN has good performance in detecting abnormalities from one-dimensional signals like EEG [47] and speech [73].
- Gait analysis, on the other hand, seems to perform better with either hybrid model (CNN-LSTM), DNN, or LSTM model. However, more research is required to determine the best-performing model.
- Apart from CNN model, Ozsahin et al. [40] and Ali et al. [75] proposed BPNN and GONN for SPECT and speech analysis respectively and obtained the highest prediction accuracy.
- However clinical trials are required to prove the suitability of the proposed deep learning model for each modality.

### 4.2. Challenges Faced by CAD Tools in Healthcare Adoption

Despite the high prediction accuracy obtained by many deep learning models proposed in various automated PD detection studies, the adoption of the deep learning model as a CAD tool in healthcare is currently not supported [21,22]. In their current form, neither neurologists nor other healthcare workers are comfortable to rely on CAD tools to diagnose the PD. This is due to several challenges as listed below:

- Lack of standards

The diagnosis of PD have been reliant on clinical features for several years, and neurologist have been trained to recognize the sets of clinical features to determine a diagnosis [8]. For instance, the diagnosis criteria provided by UKPDSBB (i.e., presence of bradykinesia and absence of certain exclusion criteria), is not adopted by current deep learning, and even machine learning studies. Instead, a majority of the deep learning studies in this review have focused on only one modality instead of adopting a multimodal approach, which is not practical for clinical use. Deep learning models also do not recognize the features of PD the same way as a human neurologist would do. For example, deep learning models can detect PD from brain imaging by means of a vectorized image instead of a clinical feature, which does not follow the existing diagnosis criteria [80]. Hence, neurologists may be too hesitant to use the CAD tools which greatly deviates from their comfort zone or does not provide a clinically trusted artificial intelligence framework that is also explainable and interpretable for future clinical practice purposes.

- Poor interpretability

Deep learning models are also known as the ‘black box’ so it is almost impossible to clearly understand the mechanisms behind a deep learning model when it makes a given prediction [22,23]. Despite achieving high prediction accuracy, end-users of the CAD tools (e.g., neurologists and healthcare workers) cannot make a diagnosis without sufficient evidence, and this evidence is not currently provided by deep learning models [21,23]. Hence, neurologists are not able to trust the CAD tools as they cannot afford to make a diagnosis without concrete evidence, explainability and interpretability of the somewhat black box style method used to produce an outcome.

- Psychological barriers

In healthcare industry, human behavior must always be considered when designing a CAD tool for a target consumer audience. The common psychological barriers that are affecting the adoption of new technologies are the endowment effect and the status quo bias. The endowment effect is where an individual values their possessions higher than their original market value [81] whereas the status quo bias is the preference of an individual to remain in their comfort zone and maintain their environment in the same state [82]. Both of these emotional biases are likely to cause an individual, neurologist, for example, to feel a significant sense of loss when they switch from manual diagnosis to relying on CAD tool for diagnosis. 

There are many other factors such as the difficulty of obtaining regulatory approval and poor interoperability, which refers to the ability to communicate between two systems [22]. For example, if two hospitals used different electronic health systems, the data from these two hospitals may not be coherent and might not communicate with each other. These two concerns, however, should come after a prototype for the CAD tool has been developed. For instance, a developer must first develop a working prototype before applying for the necessary International Organization for Standardization certifications. At present, research on using the deep learning model as a CAD tool has yet to attract end-users, and to further convince them to support the implementation of CAD tools in healthcare systems. As such, researchers must tackle the three main challenges listed above and improve the versatility of existing deep learning models. Only when the end-users are satisfied with the outcome (i.e., explainability) and the benefits (i.e., accuracy of feature extraction) of the CAD tool, they may become more willing to support the adoption of the CAD tool in healthcare. In the absence of this perceived requirement, research into a CAD-based tool for automated detection of PD and even some of the other diseases may continue to result in the ‘valley of death’, where applied research accumulates without being translated into real clinical practice. This can leading to a widening of the gap between applied research and translation of its benefits into clinical practice [83]. 

### 4.3. Solutions to Promote Adoption of CAD

**Figure 15:**
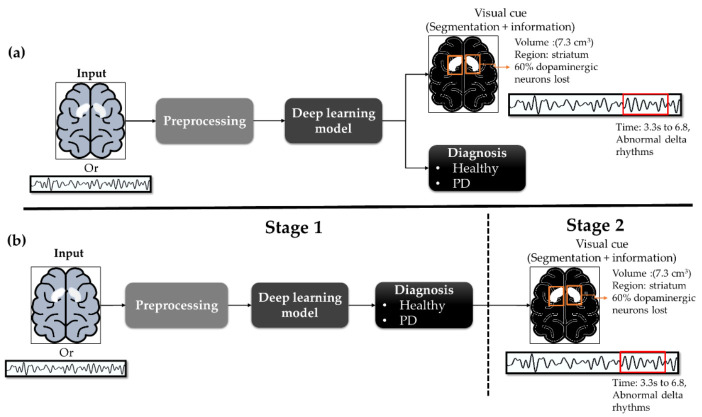
(**a**) configure a deep learning model that can perform the diagnosis (i.e., identification of the ailment) and seg-mentation (i.e., explanation, or detailed information) simultaneously; (**b**) perform diagnosis in the first stage, and in the second stage, segmentation is performed only on the input image or signal that had been diagnosed as PD in the first stage.

Moving forward with an aim to translate the potential benefits of deep learning methods into future clinical practices, researchers and end-users need to better understand that the CAD-based tool should not position itself to replace an end-user’s role in diagnosing the disease. This is a common misunderstanding as deep learning and machine learning studies often claim high success of their proposed models with the absence of end-user involvement. Consequently, a false notion of CAD tool replacing the end-users is created. Therefore, the CAD tool should aim to provide alternatives and better opinions in the diagnosis of disease for the end-users to consider, thereby increasing the end-user’s confidence and used for reducing errors simultaneously. The adoption of CAD tool, hence, should improve the efficiency of clinical diagnosis and to further help predict the possible disease and identify alternative treatment options for end-users like clinicians to consider in their days to day work. However, it appears too often that both deep learning and machine learning models do not provide additional information other than their predicted results so this may not be helpful to the end-users as a futuristic prediction tool that is not supported by visible clinical features, nor by detailed explanation of how it arrived at the results. Hence, the authors of future deep learning studies used for automated PD detection, and also for the other disease should include visual cues, such as segmentation as an explanatory function in their deep learning architecture. An example of the workflow process that we propose for a practical CAD tool is illustrated in Figure 15.

In Figure 15, we present two alternatives. The first alternative is to configure a deep learning model that can perform the diagnosis (i.e., identification of the ailment) and segmentation (i.e., explanation, or detailed information) simultaneously. The second alternative is to perform diagnosis in the first stage, and in the second stage, segmentation is performed only on the input image or signal that had been diagnosed as PD in the first stage. In either case, it will be useful to provide additional information like the time frame for abnormal physiological signals, striatal volume, and percentage of dopaminergic neurons lost for image analysis. Also, deep learning models and even machine learning models are comprised of complicated algorithms that neurologists may not necessarily understand. Hence, visual cues could make up for the poor interpretability of deep learning models by allowing neurologists to ‘see’ what has been identified as abnormalities by the model. 

**Figure 16:**
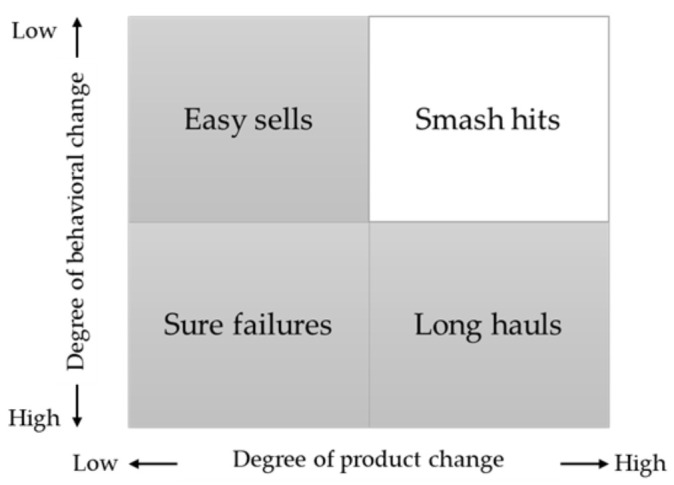
Behavioral tradeoff matrix.

The provision of visual cues may greatly contribute to the acceptance of CAD tools in healthcare. Looking at the behavioral trade-off matrix in Figure 16, innovation products are known to fall in either one of the categories [84]. At present, neurologists rely on clinical features and visual inspection to diagnose PD. However, the deep learning studies gathered in this review developed models with high prediction accuracy, but not accompanied with evidence-based diagnosis. Hence, this results in a large degree of behavioral and product change, as neurologists will have to forgo evidence-based diagnosis if they switch from visual inspection to rely on CAD tools for PD diagnosis. As a consequence, the current deep learning models developed by various study in this review falls in the ‘Sure failures’ category in Figure 16, discouraging its adoption into healthcare. The inclusion of visual cues in the deep learning model, thus, decreases the degree of behavioral change to ‘low’ as the deep learning models had segmented the brain abnormalities for the neurologist to inspect the brain images with greater ease. Also, this will greatly boost the neurologist’s confidence in deep learning models, especially when their prediction coincides with the CAD tool. Therefore, the inclusion of visual cues as a function may allow deep learning-based CAD tools to switch from the ‘Sure failures’ category to ‘Smash hits’, which greatly encourage the adoption of CAD tools and ensures the long-term and short-term success of an innovative product [84]. 

### 4.4. Solutions to Promote Adoption of CAD

**Figure 17:**
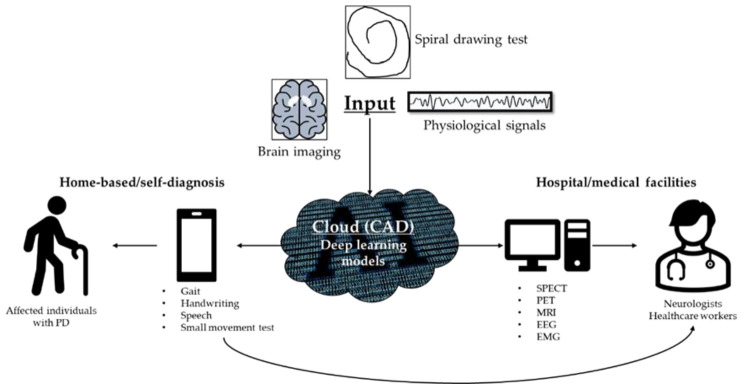
Block diagram of a Cloud-based system for PD diagnosis using various types of inputs from different modalities.

With the acceptance of the CAD-based tool, the authors hope to alleviate the manualized work burden of neurologists and other healthcare workers. As such, individuals affected by PD can also play a part by performing self-assessment with the aid of a CAD tool. This could also encourage individuals to seek professional help when the CAD tool predicted a positive on PD and urge that medical attention is required. Figure 17 is an example of a cloud-based CAD tool in which data can be assessed by any electronic device with access to the internet like smartphones and computers. An individual who suspects that they may have PD can either use their smartphone to conduct handwriting test, voice recording to detect speech aberration, or take a video of their walking cycle to perform gait analysis. These recorded pieces of evidence are useful information for the neurologist to confirm a diagnosis, which helps to increase efficiency and reduce the waiting time for diagnosis. In addition, handwriting, speech, and gait analysis are potential telemonitoring alternatives. Brain imaging like SPECT, PET, and MRI is heavy machinery that is not practical to be placed at home. Recording devices to monitor physiological signals like EEG and EMG are not common possessions in today’s households either. Hence, it is more practical to monitor PD progression thru a smartphone that has built-in handwriting, speech, and video recording function.

In this review, the authors have only demonstrated that deep learning models are promising CAD tools for PD diagnosis. However, a practical CAD tool should ideally be able to identify multiple diseases instead of PD alone. Hence, we hope deep learning studies for other neurological diseases could also heed our advice and include visual cues as a function in their system. As such, we can develop deep learning models into a clinically trusted CAD tool for clinical decision support. Thereby taking deep learning models a step further into adoption in healthcare and enter a new phase of application in the health informatics industry. 

### 4.5. Limitation of This Study

In spite of major contributions made through a detailed synthesis of the most relevant information on deep learning methods for clinical diagnosis purposes, this review comes with some limitations, as follows.

- Deep learning studies for each modality (MRI, EEG, speech, etc.,), may use different datasets to train their model. For example, studies interested in MRI may use a private dataset instead of the public dataset, PPMI. Hence, it could become rather difficult to compare the performance of two deep learning models that do not train with the same dataset.
- There is a potential lack of studies for ultrasound imaging, small movement-related tests, and multi-model analysis which involves more than one modality. This makes it difficult to determine the best-performing model for these three categories.
- The wide variety of deep learning models proposed for gait analysis also makes it challenging to determine the best performing model, hence, it is difficult to decide between the top three best performing models: CNN-LSTM, DNN, and LSTM.

## 5. Conclusions

PD requires early diagnosis and intervention to minimize the impact of this degenerative condition and ensure that affected individuals can remain self-sufficient as long as possible. However, the imprecise nature of clinical diagnoses, and a lack of neurologists expert in PD diagnosis worldwide, often results in delayed diagnosis and suboptimal management of PD. Moreover, the likely success of advanced therapeutics such as gene therapy, currently under development, will be heavily influenced by early diagnosis. Thus, a CAD tools based on deep learning models should be considered to alleviate the work burden of neurologists if they can perform fast and accurate PD diagnoses. In this study, we have reviewed 63 studies on deep learning for various modalities such as brain analysis (SPECT, PET, MRI, and EEG) and motion symptoms (gait, handwriting, speech, EMG). We show that deep learning models can achieve high prediction accuracy for PD, especially the CNN model that is widely proposed by studies that had focused on image classification for brain imaging and handwriting analysis. The CNN model also performed well in one-dimensional signals like EEG and speech analysis. However, deep learning models have yet to be supported by end-users such as neurologists and other clinicians due to a lack of evidence regarding disease prediction. Hence, this review aims to propose new solutions for future deep learning studies, and perhaps the inclusion of visual cues, such as the segmentation of abnormal areas, as a function in the deep learning model architecture. We also urge that researchers continue to build deep learning models with specific applications to some of the other disease detection problems and include visual cues in their model. It is hoped that researchers will be encouraged to adopt more explainable and interpretable methods in deep learning-based CAD tools, which can then be taken up by the end-users, and improve the health care outcomes for a growing number of individuals affected by PD worldwide. 

## Figures and Tables

**Table 1:** Summary of the Boolean search string across the respective journal article databases.

	Boolean Search String		
Database	[Title]	AND [Title/Abstract]	No. of Studies
PubMed	“parkinson” AND “disease”	“Neural network”	178
		“Deep learning”	
Google Scholar		“Prediction” OR “Diagnosis” OR “Detection”	248
IEEE		“Neural network”	135
		“Deep learning”	
Science direct		“Neural network”	233
		“Deep learning”	

## Data Availability

Not applicable.

## Appendix A

**Table A1:** List of deep learning studies for various modalities in brain analysis.

Year	Author	Input Feature	Approach	Dataset	Accuracy (%)
**MRI**					
2019	Xiao et al. [85]	Quantitative susceptibility mapping (QSM) images	CNN	87 PD; 53 HC (private)	89.0
2021	Yasaka et al. [86]	radial kurtosis (RK) Connectrome matrix	CNN	115 PD; 115 HC (private)	81.0
2020	Chakraborty et al. [42]	Normalized MRI images	CNN	203 PD; 203 HC (PPMI)	95.3
2020	Tremblay et al. [87]	T2-weighted imaging	CNN	15 PD; 15 HC (private)	88.3
2019	Shinde et al. [88]	a boxed region around the brain-stem on the axial slices of the NMS-MRI as input	CNN (ResNet50)	45 PD; 35 HC (private)	80.0
**PET/CT**					
2021	Piccardo et al. [41]	[18F]DOPA PET/CT images	CNN (3D)	43 PD; 55 HC (private)	93.0
2019	Shen et al. [89]	laconic representation of PET images	Group Lasso Sparse Deep Belief Network (GLS-DBN)	125 PD; 225 HC (private)	90.0
2019	Dai et al. [90]	Enhanced Pet images	CNN (U-net)	214 PD; 127 HC (PPMI)	84.2
**SPECT/DaTscan**					
2015	Hirschauer et al. [91]	Inputs from all 8 diagnostic test in database	Enhanced probabilistic neural network (EPNN)	189 PD; 415 HC (PPMI)	98.6
2020	Ozsahin et al. [40]	Binarized images	back propagation neural network (BPNN)	1.334 PD; 212 HC (PPMI)	99.6
2017	Choi et al. [37]	Normalized SPECT images	CNN	431 PD; 193 HC (combination of 2 database)	98.8
2020	Magesh et al. [92]	Normalized SPECT images	CNN (VGG16)	430 PD; 212 HC (PPMI)	95.2
2020	Chien et al. [93]	segmented striatal region images	CNN	234 PD; 145 HC (private)	86.0
2020	Hsu et al. [94]	Grayscale + colour SPECT images	CNN (VGG)	196 PD; 6 HC (private)	85.0
2019	Ortiz et al. [95]	Voxel feature extracted via isosurfaces	CNN (3D)	158 PD; 111 HC (PPMI)	95.1
2018	Martinez-Murcia at al. [96]	Normalized DaTSCAN images	CNN (ALEXNET)	448 PD; 194 HC (PPMI)	94.1
**Ultrasound**					
2018	Shen et al. [43]	73 features extracted from Transcranial sonography (TCS) image	MEKM-BLS	76 PD; 77HC (private)	78.4
**EEG**					
2020	Xu et al. [49]	end-to-end EEG signals	pooling-based deep recurrent neural network (PDRNN)	10 PD; 10 HC (private)	88.6
2021	Lee et al. [51]	spatiotemporal features of EEG signals	CRNN	-	99.2
2021	Loh et al. [25]	Spectrograms images	CNN	15 PD; 16 HC (public)	99.5
2021	Khare et al. [47]	smoothed pseudo-Wigner Ville distribution	CNN	15 PD; 16 HC (public)	100
2018	Oh et al. [48]	end-to-end EEG signals	13-layer 1D-CNN	20 PD; 20 HC (private)	88.3
2020	Shah et al. [50]	-	DNN	-	99.2

**Table A2:** List of deep learning studies for various modalities in motor symptoms.

Year	Author	Input Feature	Approach	Dataset	Accuracy (%)
**Gait**					
2019	Xia et al. [53]	Multi-points Vertical Ground Reaction Force (VGRF) time series	CNN-LSTM	93 PD; 73 HC (public)	99.1
2016	Nancy Jane et al. [97]	Temporal sequence of walking pattern	Q-BTDNN	93 PD; 73 HC (public)	93.1 [Ga]91.7 [Si]89.7 [Ju]
2020	Som et al. [98]	Reduced feature via PCA	Autoencoder	18 PD; 16 HC (public)	73.8
2020	Zhang et al. [99]	Normalization and Data Augmentation	CNN	656 PD; 2148 HC (public)	86.0
2020	Maachi et al. [58]	18 1D-signals	DNN	93 PD; 73 HC (public)	98.7
2021	Balaji et al. [59]	the gait kinematic features	LSTM	-	98.6
2020	Yurdakul et al. [100]	NR-LBP	ANN	93 PD; 73 HC (public)	98.3
2018	Zhao et al. [54]	19 features	CNN-LSTM	93PD; 73 HC (public)	98.0
2016	Zeng et al. [101]	19 features	RBF-NN	93PD; 73 HC (public)	96.4
2020	Alharthi et al. [102]	ground reaction force	CNN	93 PD; 73 HC (public)	95.5
2020	Butt et al. [103]	kinematic features	LSTM	64 PD; 50 HC (private)	82.4
**Handwriting**					
2021	Folador et al. [104]	histograms of oriented gradients (HOG)	CNN	20 PD; 20 HC	83.1
2019	Yang et al. [105]	key parameters deviation (cm) and accumulation angle (rad)	GRNN	21 PD; 24 HC	98.9
2020	Canturk et al. [106]	Fuzzy recurrence plot (FRP)	CNN	25 PD; 15 HC	94.0
2019	Gil-Martín et al. [107]	CNN based features	CNN	62 PD; 15 HC	96.5
2019	Naseer et al. [108]	CNN based features	CNN	37 PD; 38 HC	98.3
2021	Gazda et al. [109]	handwriting images	CNN	-	94.7
2020	Kamran et al. [65]	CNN based features	CNN	PaHaW dataset[38/37], HandPD dataset[18/74], NewHandPD dataset[35/31] Parkinsons Drawing	99.2
2018	Pereira et al. [110]	CNN based features	CNN	74 PC; 18 HC	95.0
2018	Afonso et al. [111]	recurrence plots to map the signals onto the image domain	CNN	14 PD; 21 HC	87.0
2019	Ribeiro et al. [112]	Bags of Sampling	RNN	14 PD; 21 HC	97.0
2019	Diaz et al. [113]	Generate enhanced images	CNN	37 PD; 38 HC	86.67
2021	Diaz et al. [114]	Kinematic and pressure features	CNN-RNN	PaHaW dataset[38/37], NewHandPD dataset[35/31]	90.0
2020	Nomm et al. [115]	Image of a drawn spiral enhanced by the velocity and pressure parameters	CNN	17 PD; 17 HC	93
**Movement**					
2018	Prince et al. [71]	touch-screen and accelerometer waveforms	CNN	949 PD; 866 HC	62.1
2017	Jones et al. [70]	Temporal Manometric and videofluoroscopic data	ANN	31 PD; 31 HC	82.3
**Speech**					
2018	Putri et al. [76]	Various voice measurements	ANN	15 PD; 8 HC	94.4
2019	Ali et al. [75]	dimensionality reduction of all 26 features by LDA	GONN	20 PD; 20 HC (Sakar, 2013)	100
2015	Peker et al. [116]	12 features selected by minimum redundancy maximum relevance (mRMR) attribute selection algorithm	CVANN	23 PD; 8 HC (Little, 2007)	98.1
2019	Wodzinski et al. [117]	Spectrograms images	CNN	50 PD; 50 HC (PC-GITA)	91.7
2016	Avci et al. [118]	22 biomedical voice measurements	ELM	23 PD; 8 HC (Little, 2007)	96.8
2017	Gómez-Vilda et al. [119]	absolute kinematic velocity (AKV) distribution	RLSFN	53 PD; 26 HC (Male) 38 PD; 25 HC (Female)	99.4
2020	Nagasubramanian et al. [73]	All 26 features	CNN	20 PD; 20 HC (Sakar, 2013)	99.5
2020	Xu et al. [120]	Spectrograms images	CNN	20 PD; 20 HC (Sakar, 2013)	91.2
2021	Karaman et al. [121]	CNN based features	CNN	mPower Voice database	91.17
2011	Åström et al. [122]	10 vocal features	DNN	23 PD; 8 HC (Little, 2007)	91.2
2021	Narendra et al. [123]	raw speech and voice source waveforms	CNN	50 PD; 50 HC (PC-GITA)	68.6
2021	Goyal et al. [74]	A combination of Resonance based Sparse Signal Decomposition (RSSD) + Time-Frequency (T-F) algorithm	CNN	16 PD; 21 HC and 20 HC	99.4
**EMG**					
2018	Putri et al. [76]	12 EMG features	ANN	15 PD; 8 HC	71.0
**Mixture of inputs**					
2018	Vasquez-Correa et al. [77]	Spectrograms images	CNN	44 PD; 40 HC	97.6
2017	Oung et al. [78]	Empirical Wavelet Transform Based Features	ELM	50 PD; 15 HC	95.93

## References

[1] Politis M., Wu K., Molloy S., Bain P.G., Chaudhuri K.R., Piccini P. (2010). Parkinson’s disease symptoms: The patient’s perspective. Mov. Disord..

[2] Balestrino R., Schapira A.H.V. (2020). Parkinson disease. Eur. J. Neurol..

[3] Bhat S., Acharya U.R., Hagiwara Y., Dadmehr N., Adeli H. (2018). Parkinson’s disease: Cause factors, measurable indicators, and early diagnosis. Comput. Biol. Med..

[4] Dorsey E.R., Elbaz A., Nichols E., Abd-Allah F., Abdelalim A., Adsuar J.C., Ansha M.G., Brayne C., Choi J.-Y.J., Collado-Mateo D. (2018). Global, regional, and national burden of Parkinson’s disease, 1990–2016: A systematic analysis for the Global Burden of Disease Study 2016. Lancet Neurol..

[5] Bloem B.R., Okun M.S., Klein C. (2021). Parkinson’s disease. Lancet.

[6] Szász J.A., Orbán-Kis K., Constantin V.A., Péter C., Bíró I., Mihály I., Szegedi K., Balla A., Szatmári S. (2019). Therapeutic strategies in the early stages of Parkinson’s disease: A cross-sectional evaluation of 15 years’ experience with a large cohort of Romanian patients. Neuropsychiatr. Dis. Treat..

[7] Dangouloff T., Servais L. (2019). Clinical evidence supporting early treatment of patients with spinal muscular atrophy: Current perspectives. Ther. Clin. Risk Manag..

[8] Berardelli A., Wenning G.K., Antonini A., Berg D., Bloem B.R., Bonifati V., Brooks D., Burn D.J., Colosimo C., Fanciulli A. (2013). EFNS/MDS-ES recommendations for the diagnosis of Parkinson’s disease. Eur. J. Neurol..

[9] Rizzo G., Copetti M., Arcuti S., Martino D., Fontana A., Logroscino G. (2016). Accuracy of clinical diagnosis of Parkinson disease. Neurology.

[10] Burton A. (2018). How do we fix the shortage of neurologists?. Lancet Neurol..

[11] Segato A., Marzullo A., Calimeri F., De Momi E. (2020). Artificial intelligence for brain diseases: A systematic review. APL Bioeng..

[12] Raghavendra U., Acharya U.R., Adeli H. (2019). Artificial Intelligence techniques for automated diagnosis of neurological disorders. Eur. Neurol..

[13] Yuvaraj R., Murugappan M., Acharya U.R., Adeli H., Ibrahim N.M., Mesquita E. (2016). Brain functional connectivity patterns for emotional state classification in Parkinson’s disease patients without dementia. Behav. Brain Res..

[14] Tuncer T., Dogan S., Acharya U.R. (2020). Automated detection of Parkinson’s disease using minimum average maximum tree and singular value decomposition method with vowels. Biocybern. Biomed. Eng..

[15] Faust O., Razaghi H., Barika R., Ciaccio E.J., Acharya U.R. (2019). A review of automated sleep stage scoring based on physiological signals for the new millennia. Comput. Methods Programs Biomed..

[16] Loh H.W., Ooi C.P., Vicnesh J., Oh S.L., Faust O., Gertych A., Acharya U.R. (2020). Automated detection of sleep stages using deep learning techniques: A systematic review of the last decade (2010–2020). Appl. Sci..

[17] Khare S.K., Bajaj V., Acharya U.R. (2021). Detection of Parkinson’s disease using automated tunable Q wavelet transform technique with EEG signals. Biocybern. Biomed. Eng..

[18] Bhurane A.A., Dhok S., Sharma M., Yuvaraj R., Murugappan M., Acharya U.R. (2019). Diagnosis of Parkinson’s disease from electroencephalography signals using linear and self-similarity features. Expert Syst..

[19] Yuvaraj R., Rajendra Acharya U.R., Hagiwara Y. (2018). A novel Parkinson’s disease diagnosis index using higher-order spectra features in EEG signals. Neural Comput. Appl..

[20] Mirza B., Wang W., Wang J., Choi H., Chung N.C., Ping P. (2019). Machine learning and integrative analysis of biomedical big data. Genes.

[21] Taylor J., Fenner J. (2019). The challenge of clinical adoption—The insurmountable obstacle that will stop machine learning?. BJR Open.

[22] Varghese J. (2020). Artificial intelligence in medicine: Chances and challenges for wide clinical adoption. Visc. Med..

[23] Lee J.-G., Jun S., Cho Y.-W., Lee H., Kim G.B., Seo J.B., Kim N. (2017). Deep learning in medical imaging: General overview. Korean J. Radiol..

[24] Balderas Silva D., Ponce Cruz P., Molina Gutierrez A. (2018). Are the long–short term memory and convolution neural networks really based on biological systems?. ICT Express.

[25] Loh H., Ooi C., Palmer E., Barua P., Dogan S., Tuncer T., Baygin M., Acharya U. (2021). GaborPDNet: Gabor transformation and deep neural network for Parkinson’s disease detection using EEG signals. Electronics.

[26] Sarvamangala D.R., Kulkarni R.V. (2021). Convolutional neural networks in medical image understanding: A survey. Evol. Intell..

[27] Fan J., Xu W., Wu Y., Gong Y. (2010). Human tracking using convolutional neural networks. IEEE Trans. Neural Netw..

[28] Lu J., Liong V.E., Wang G., Moulin P. (2015). Joint feature learning for face recognition. IEEE Trans. Inf. Forensics Secur..

[29] Hochreiter S., Schmidhuber J. (1997). Long short-term memory. Neural Comput..

[30] Jiang C., Chen Y., Chen S., Bo Y., Li W., Tian W., Jun G. (2019). A mixed deep recurrent neural network for MEMS gyroscope noise suppressing. Electronics.

[31] Gers F.A., Schmidhuber J., Cummins F. (2000). Learning to forget: Continual prediction with LSTM. Neural Comput..

[32] Coto-Jiménez M. (2019). Improving post-filtering of artificial speech using pre-trained LSTM neural networks. Biomimetics.

[33] Graves A., Liwicki M., Fernandez S., Bertolami R., Bunke H., Schmidhuber J. (2009). A Novel connectionist system for unconstrained handwriting recognition. IEEE Trans. Pattern Anal. Mach. Intell..

[34] Nabipour M., Nayyeri P., Jabani H., Mosavi A., Salwana E., Shahab S. (2020). Deep learning for stock market prediction. Entropy.

[35] Qiu J., Wang B., Zhou C. (2020). Forecasting stock prices with long-short term memory neural network based on attention mechanism. PLoS ONE.

[36] Moher D., Liberati A., Tetzlaff J., Altman D.G. (2009). Preferred reporting items for systematic reviews and meta-analyses: The PRISMA statement. PLoS Med..

[37] Choi H., Ha S., Im H.J., Paek S.H., Lee D.S. (2017). Refining diagnosis of Parkinson’s disease with deep learning-based interpretation of dopamine transporter imaging. NeuroImage Clin..

[38] Garibotto V., Montandon M.L., Viaud C.T., Allaoua M., Assal F., Burkhard P.R., Ratib O., Zaidi H. (2013). Regions of interest–based discriminant analysis of DaTSCAN SPECT and FDG-PET for the classification of dementia. Clin. Nucl. Med..

[39] Meyer P.T., Frings L., Rücker G., Hellwig S. (2017). 18 F-FDG PET in Parkinsonism: Differential diagnosis and evaluation of cognitive impairment. J. Nucl. Med..

[40] Ozsahin I., Sekeroglu B., Pwavodi P.C., Mok G.S.P. (2020). High-accuracy automated diagnosis of Parkinson’s disease. Curr. Med. Imaging Former. Curr. Med. Imaging Rev..

[41] Piccardo A., Cappuccio R., Bottoni G., Cecchin D., Mazzella L., Cirone A., Righi S., Ugolini M., Bianchi P., Bertolaccini P. (2021). The role of the deep convolutional neural network as an aid to interpreting brain [18F]DOPA PET/CT in the diagnosis of Parkinson’s disease. Eur. Radiol..

[42] Chakraborty S., Aich S., Kim H.-C. (2020). Detection of Parkinson’s disease from 3T T1 weighted MRI scans using 3D convolutional neural network. Diagnostics.

[43] Shen L., Shi J., Gong B., Zhang Y., Dong Y., Zhang Q., An H. Multiple empirical kernel mapping based broad learning system for classification of Parkinson’s disease with transcranial sonography. Proceedings of the 40th Annual International Conference of the IEEE Engineering in Medicine and Biology Society (EMBC).

[44] Mehnert S., Reuter I., Schepp K., Maaser P., Stolz E., Kaps M. (2010). Transcranial sonography for diagnosis of Parkinson’s disease. BMC Neurol..

[45] Barua P.D., Dogan S., Tuncer T., Baygin M., Acharya U.R. (2021). Novel automated PD detection system using aspirin pattern with EEG signals. Comput. Biol. Med..

[46] Soikkeli R., Partanen J., Soininen H., Pääkkönen A., Riekkinen P. (1991). Slowing of EEG in Parkinson’s disease. Electroencephalogr. Clin. Neurophysiol..

[47] Khare S.K., Bajaj V., Acharya U.R. (2021). PDCNNet: An automatic framework for the detection of Parkinson’s disease using EEG signals. IEEE Sens. J..

[48] Oh S.L., Hagiwara Y., Raghavendra U., Yuvaraj R., Arunkumar N., Murugappan M., Acharya U.R. (2020). A deep learning approach for Parkinson’s disease diagnosis from EEG signals. Neural Comput. Appl..

[49] Xu S., Wang Z., Sun J., Zhang Z., Wu Z., Yang T., Xue G., Cheng C. (2020). Using a deep recurrent neural network with EEG signal to detect Parkinson’s disease. Ann. Transl. Med..

[50] Shah S.A.A., Zhang L., Bais A. (2020). Dynamical system based compact deep hybrid network for classification of Parkinson disease related EEG signals. Neural Netw..

[51] Lee S., Hussein R., Ward R., Jane Wang Z., McKeown M.J. (2021). A convolutional-recurrent neural network approach to resting-state EEG classification in Parkinson’s disease. J. Neurosci. Methods.

[52] Di Biase L., Di Santo A., Caminiti M.L., De Liso A., Shah S.A., Ricci L., Di Lazzaro V. (2020). Gait analysis in Parkinson’s disease: An overview of the most accurate markers for diagnosis and symptoms monitoring. Sensors.

[53] Xia Y., Yao Z., Ye Q., Cheng N. (2020). A dual-modal attention-enhanced deep learning network for quantification of Parkinson’s disease characteristics. IEEE Trans. Neural Syst. Rehabil. Eng..

[54] Zhao A., Qi L., Li J., Dong J., Yu H. (2018). A hybrid spatio-temporal model for detection and severity rating of Parkinson’s disease from gait data. Neurocomputing.

[55] Yogev G., Giladi N., Peretz C., Springer S., Simon E.S., Hausdorff J.M. (2005). Dual tasking, gait rhythmicity, and Parkinson’s disease: Which aspects of gait are attention demanding?. Eur. J. Neurosci..

[56] Hausdorff J.M., Lowenthal J., Herman T., Gruendlinger L., Peretz C., Giladi N. (2007). Rhythmic auditory stimulation modulates gait variability in Parkinson’s disease. Eur. J. Neurosci..

[57] Frenkel-Toledo S., Giladi N., Peretz C., Herman T., Gruendlinger L., Hausdorff J.M. (2005). Treadmill walking as an external pacemaker to improve gait rhythm and stability in Parkinson’s disease. Mov. Disord..

[58] El Maachi I., Bilodeau G.-A., Bouachir W. (2020). Deep 1D-Convnet for accurate Parkinson disease detection and severity prediction from gait. Expert Syst. Appl..

[59] Balaji E., Brindha D., Elumalai V.K., Vikrama R. (2021). Automatic and non-invasive Parkinson’s disease diagnosis and severity rating using LSTM network. Appl. Soft Comput..

[60] Thomas M., Lenka A., Kumar Pal P. (2017). Handwriting analysis in Parkinson’s disease: Current status and future directions. Mov. Disord. Clin. Pract..

[61] McLennan J.E., Nakano K., Tyler H.R., Schwab R.S. (1972). Micrographia in Parkinson’s disease. J. Neurol. Sci..

[62] Drotár P., Mekyska J., Rektorová I., Masarová L., Smékal Z., Faundez-Zanuy M. (2016). Evaluation of handwriting kinematics and pressure for differential diagnosis of Parkinson’s disease. Artif. Intell. Med..

[63] Pereira C.R., Pereira D.R., Silva F.A., Masieiro J.P., Weber S.A.T., Hook C., Papa J.P. (2016). A new computer vision-based approach to aid the diagnosis of Parkinson’s disease. Comput. Methods Programs Biomed..

[64] Pereira C.R., Weber S.A.T., Hook C., Rosa G.H., Papa J.P. Deep learning-aided Parkinson’s disease diagnosis from handwritten dynamics. Proceedings of the 29th SIBGRAPI Conference on Graphics, Patterns and Images (SIBGRAPI).

[65] Kamran I., Naz S., Razzak I., Imran M. (2021). Handwriting dynamics assessment using deep neural network for early identification of Parkinson’s disease. Future Gener. Comput. Syst..

[66] Krizhevsky A., Sutskever I., Hinton G.E. (2017). ImageNet classification with deep convolutional neural networks. Commun. ACM.

[67] Szegedy C., Liu W., Jia Y., Sermanet P., Reed S., Anguelov D., Erhan D., Vanhoucke V., Rabinovich A. Going deeper with convolutions. Proceedings of the 28th IEEE Conference on Computer Vision and Pattern Recognition (CVPR).

[68] Simonyan K., Zisserman A. (2014). Very deep convolutional networks for large-scale image recognition. arXiv.

[69] He K., Zhang X., Ren S., Sun J. Deep residual learning for image recognition. Proceedings of the 2016 IEEE Conference on Computer Vision and Pattern Recognition (CVPR).

[70] Jones C.A., Hoffman M.R., Lin L., Abdelhalim S., Jiang J.J., McCulloch T.M. (2018). Identification of swallowing disorders in early and mid-stage Parkinson’s disease using pattern recognition of pharyngeal high-resolution manometry data. Neurogastroenterol. Motil..

[71] Prince J., de Vos M. A deep learning framework for the remote detection of Parkinson’S Disease using smart-phone sensor data. Proceedings of the 40th Annual International Conference of the IEEE Engineering in Medicine and Biology Society (EMBC).

[72] Tjaden K. (2008). Speech and swallowing in Parkinson’s disease. Top. Geriatr. Rehabil..

[73] Nagasubramanian G., Sankayya M. (2021). Multi-variate vocal data analysis for detection of Parkinson disease using deep learning. Neural Comput. Appl..

[74] Goyal J., Khandnor P., Aseri T.C. (2021). A hybrid approach for Parkinson’s disease diagnosis with resonance and time-frequency based features from speech signals. Expert Syst. Appl..

[75] Ali L., Zhu C., Zhang Z., Liu Y. (2019). Automated Detection of Parkinson’s disease based on multiple types of sustained phonations using linear discriminant analysis and genetically optimized neural network. IEEE J. Transl. Eng. Health Med..

[76] Putri F., Caesarendra W., Pamanasari E.D., Ariyanto M., Setiawan J.D. (2018). Parkinson disease detection based on voice and EMG pattern classification method for Indonesian case study. J. Energy Mech. Mater. Manuf. Eng..

[77] Vasquez-Correa J.C., Arias-Vergara T., Orozco-Arroyave J.R., Eskofier B., Klucken J., Noth E. (2019). Multimodal assessment of Parkinson’s disease: A deep learning approach. IEEE J. Biomed. Health Inform..

[78] Oung Q.W., Muthusamy H., Basah S.N., Lee H., Vijean V. (2018). Empirical Wavelet transform based features for classification of Parkinson’s disease severity. J. Med. Syst..

[79] Ding S., Zhao H., Zhang Y., Xu X., Nie R. (2015). Extreme learning machine: Algorithm, theory and applications. Artif. Intell. Rev..

[80] Panch T., Mattie H., Celi L.A. (2019). The ‘inconvenient truth’ about AI in healthcare. NPJ Digit. Med..

[81] Melnychenko O. (2020). Is artificial intelligence ready to assess an enterprise’s financial security?. J. Risk Financ. Manag..

[82] Tavares J., Ong F.S., Ye T., Xue J., He M., Gu J., Lin H., Xu B., Cheng Y. (2019). Psychosocial factors affecting artificial intelligence adoption in health care in China: Cross-sectional study. J. Med. Internet Res..

[83] Butler D. (2008). Translational research: Crossing the valley of death. Nature.

[84] Gourville J.T. (2006). Eager sellers and stony buyers: Understanding the psychology of new-product adoption. Harv. Bus. Rev..

[85] Xiao B., He N., Wang Q., Cheng Z., Jiao Y., Haacke E.M., Yan F., Shi F. (2019). Quantitative susceptibility mapping based hybrid feature extraction for diagnosis of Parkinson’s disease. NeuroImage Clin..

[86] Yasaka K., Kamagata K., Ogawa T., Hatano T., Takeshige-Amano H., Ogaki K., Andica C., Akai H., Kunimatsu A., Uchida W. (2021). Parkinson’s disease: Deep learning with a parameter-weighted structural connectome matrix for diagnosis and neural circuit disorder investigation. Neuroradiology.

[87] Tremblay C., Mei J., Frasnelli J. (2020). Olfactory bulb surroundings can help to distinguish Parkinson’s disease from non-parkinsonian olfactory dysfunction. NeuroImage Clin..

[88] Shinde S., Prasad S., Saboo Y., Kaushick R., Saini J., Pal P.K., Ingalhalikar M. (2019). Predictive markers for Parkinson’s disease using deep neural nets on neuromelanin sensitive MRI. NeuroImage Clin..

[89] Shen T., Jiang J., Lin W., Ge J., Wu P., Zhou Y., Zuo C., Wang J., Yan Z., Shi K. (2019). Use of overlapping group LASSO sparse deep belief network to discriminate Parkinson’s disease and normal control. Front. Neurosci..

[90] Dai Y., Tang Z., Wang Y., Xu Z. (2019). Data driven intelligent diagnostics for Parkinson’s disease. IEEE Access.

[91] Hirschauer T.J., Adeli H., Buford J.A. (2015). Computer-aided diagnosis of Parkinson’s disease using enhanced probabilistic neural network. J. Med. Syst..

[92] Magesh P.R., Myloth R.D., Tom R.J. (2020). An explainable machine learning model for early detection of Parkinson’s disease using LIME on DaTSCAN imagery. Comput. Biol. Med..

[93] Chien C.-Y., Hsu S.-W., Lee T.-L., Sung P.-S., Lin C.-C. (2020). Using artificial neural network to discriminate Parkinson’s disease from other Parkinsonisms by focusing on putamen of dopamine transporter SPECT images. Biomedicines.

[94] Hsu S.-Y., Yeh L.-R., Chen T.-B., Du W.-C., Huang Y.-H., Twan W.-H., Lin M.-C., Hsu Y.-H., Wu Y.-C., Chen H.-Y. (2020). Classification of the multiple stages of Parkinson’s Disease by a deep convolution neural network based on 99mTc-TRODAT-1 SPECT images. Molecules.

[95] Ortiz A., Munilla J., Martínez-Ibañez M., Górriz J.M., Ramírez J., Salas-Gonzalez D. (2019). Parkinson’s disease detection using isosurfaces-based features and convolutional neural networks. Front. Neuroinform..

[96] Martinez-Murcia F.J., Górriz J.M., Ramírez J., Ortiz A. (2018). Convolutional neural networks for neuroimaging in Parkinson’s disease: Is preprocessing needed?. Int. J. Neural Syst..

[97] Nancy Jane Y., Khanna Nehemiah H., Arputharaj K. (2016). A Q-backpropagated time delay neural network for diagnosing severity of gait disturbances in Parkinson’s disease. J. Biomed. Inform..

[98] Som A., Krishnamurthi N., Buman M., Turaga P. Unsupervised pre-trained models from healthy ADLs improve Parkinson’s disease classification of gait patterns. Proceedings of the 42nd Annual International Conference of the IEEE Engineering in Medicine & Biology Society (EMBC).

[99] Zhang H., Deng K., Li H., Albin R.L., Guan Y. (2020). Deep learning identifies digital biomarkers for self-reported Parkinson’s disease. Patterns.

[100] Yurdakul O.C., Subathra M.S.P., George S.T. (2020). detection of parkinson’s disease from gait using neighborhood representation local binary patterns. Biomed. Signal Process. Control..

[101] Zeng W., Liu F., Wang Q., Wang Y., Ma L., Zhang Y. (2016). Parkinson’s disease classification using gait analysis via deterministic learning. Neurosci. Lett..

[102] Alharthi A.S., Casson A.J., Ozanyan K.B. (2021). Gait spatiotemporal signal analysis for Parkinson’s disease detection and severity rating. IEEE Sens. J..

[103] Butt A.H., Cavallo F., Maremmani C., Rovini E. Biomechanical parameters assessment for the classification of Parkinson disease using bidirectional long short-term memory. Proceedings of the 42nd Annual International Conference of the IEEE Engineering in Medicine & Biology Society (EMBC).

[104] Folador J.P., Santos M.C.S., Luiz L.M.D., De Souza L.A.P.S., Vieira M.F., Pereira A.A., Andrade A.D.O. (2021). On the use of histograms of oriented gradients for tremor detection from sinusoidal and spiral handwritten drawings of people with Parkinson’s disease. Med. Biol. Eng. Comput..

[105] Yang T.-L., Lin C.-H., Chen W.-L., Lin H.-Y., Su C.-S., Liang C.-K. (2020). Hash transformation and machine learning-based decision-making classifier improved the accuracy rate of automated Parkinson’s disease screening. IEEE Trans. Neural Syst. Rehabil. Eng..

[106] Cantürk İ. (2021). Fuzzy recurrence plot-based analysis of dynamic and static spiral tests of Parkinson’s disease patients. Neural Comput. Appl..

[107] Gil-Martín M., Montero J.M., San-Segundo R. (2019). Parkinson’s disease detection from drawing movements using convolutional neural networks. Electronics.

[108] Naseer A., Rani M., Naz S., Razzak M.I., Imran M., Xu G. (2020). Refining Parkinson’s neurological disorder identification through deep transfer learning. Neural Comput. Appl..

[109] Gazda M., Hires M., Drotar P. (2021). Multiple-fine-tuned convolutional neural networks for Parkinson’s disease diagnosis from offline handwriting. IEEE Trans. Syst. Man Cybern. Syst..

[110] Pereira C.R., Pereira D.R., de Rosa G.H., Albuquerque V.H.C., Weber S.A., Hook C., Papa J.P. (2018). Handwritten dynamics assessment through convolutional neural networks: An application to Parkinson’s disease identification. Artif. Intell. Med..

[111] Afonso L.C., Rosa G.H., Pereira C.R., Weber S.A., Hook C., Albuquerque V.H.C., Papa J.P. (2019). A recurrence plot-based approach for Parkinson’s disease identification. Futur. Gener. Comput. Syst..

[112] Ribeiro L.C.F., Afonso L.C.S., Papa J.P. (2019). Bag of samplings for computer-assisted Parkinson’s disease diagnosis based on recurrent neural networks. Comput. Biol. Med..

[113] Diaz M., Ferrer M.A., Impedovo D., Pirlo G., Vessio G. (2019). Dynamically enhanced static handwriting representation for Parkinson’s disease detection. Pattern Recognit. Lett..

[114] Diaz M., Moetesum M., Siddiqi I., Vessio G. (2021). Sequence-based dynamic handwriting analysis for Parkinson’s disease detection with one-dimensional convolutions and BiGRUs. Expert Syst. Appl..

[115] Nõmm S., Zarembo S., Medijainen K., Taba P., Toomela A. (2020). Deep CNN Based classification of the archimedes spiral drawing tests to support diagnostics of the Parkinson’s disease. IFAC Pap.Online.

[116] Peker M., Şen B., Delen D. (2015). Computer-aided diagnosis of Parkinson’s disease using complex-valued neural networks and mRMR feature selection algorithm. J. Healthc. Eng..

[117] Wodzinski M., Skalski A., Hemmerling D., Orozco-Arroyave J.R., Noth E. Deep learning approach to Parkinson’s disease detection using voice recordings and convolutional neural network dedicated to image classification. Proceedings of the 41st Annual International Conference of the IEEE Engineering in Medicine and Biology Society (EMBC).

[118] Avci D., Dogantekin A. (2016). An expert diagnosis system for Parkinson disease based on genetic algorithm-wavelet kernel-extreme learning machine. Parkinson’s Dis..

[119] Gómez-Vilda P., Mekyska J., Ferrández J.M., Palacios-Alonso D., Gómez-Rodellar A., Rodellar-Biarge V., Galaz Z., Smekal Z., Eliasova I., Kostalova M. (2017). Parkinson disease detection from speech articulation neuromechanics. Front. Neuroinform..

[120] Xu Z.-J., Wang R.-F., Wang J., Yu D.-H. (2020). Parkinson’s disease detection based on spectrogram-deep convolutional generative adversarial network sample augmentation. IEEE Access.

[121] Karaman O., Çakın H., Alhudhaif A., Polat K. (2021). Robust automated Parkinson disease detection based on voice signals with transfer learning. Expert Syst. Appl..

[122] Åström F., Koker R. (2011). A parallel neural network approach to prediction of Parkinson’s disease. Expert Syst. Appl..

[123] Narendra N.P., Schuller B., Alku P. (2021). The detection of Parkinson’s Disease from speech using voice source information. IEEE ACM Trans. Audio Speech Lang. Process..

